# Supplementary material for: Towards a component-based system model to improve the quality of highly configurable systems
Source: PeerJ Comput Sci. 2022 Mar 7;8:e912. doi: 10.7717/peerj-cs.912 (PMC9044257; doi:10.7717/peerj-cs.912)
Supplement: Supplemental Information 1 [file peerj-cs-08-912-s001.zip › Component-Reuse-EJS-App-main/views/viewCode.ejs]

Components reuse App


<%-include('includes/\_header')%>


Back
<%if(name){%>

#### Component Found Successfully!

---

##### Component Name

<%= name%>

  

##### Component Code

```
<%= code%>
```

<%} else {%>

#### Sorry! No Component Found matching this name.

<%}%>


<%-include('includes/\_footer')%>
