# Supplementary material for: Towards a component-based system model to improve the quality of highly configurable systems
Source: PeerJ Comput Sci. 2022 Mar 7;8:e912. doi: 10.7717/peerj-cs.912 (PMC9044257; doi:10.7717/peerj-cs.912)
Supplement: Supplemental Information 2 [file peerj-cs-08-912-s002.docx]

1. **Questionnaires**

Table 0.3: Background assessment of subjects

| **Questions** | **Possible Answers** |
| --- | --- |
| Have you studied the Software product management or requirement engineering course in your academic career? | Text |
| How many software engineering projects, you were involved in? | Integer/Text |
| Any biggest achievement in your academic project? | Integer/Text |
| Any industry project where you were involved in? | Integer/Text |
| Except for academic and industry projects, any open-source projects where you were involved in? | Integer/Text |
| Did you involved any activities related to software engineering, SPM, or SDLC? | Integer/Text |
| Do you think you are Beginner/Mediate/Expert in software engineering? | Beginner/ Moderate/ Expert |
| Did you already hear something about software product line engineering and Agile development processes? | Integer/Text |

Table 0.4: Questionnaires for post experiment analysis and practioners reviews.

| **RQs** | **Questions** | **Possible Answers** |
| --- | --- | --- |
| RQ1(a) | How much it was easy to understand, execute and complete application requirements engineering process? | Likert-Scale  (1-5) |
| RQ1(b) | How much it was easy to understand, execute and complete common reference architecture? | Likert-Scale  (1-5) |
| RQ1(c) | How much it was easy to understand, execute and complete variations and commonalities identification? | Likert-Scale  (1-5) |
| RQ1(d) | How much it was easy to understand, execute and complete component selection? | Likert-Scale  (1-5) |
| RQ1(e) | How much it was easy to understand, execute and complete dependency evaluation? | Likert-Scale  (1-5) |
| RQ1(f) | How much it was easy to understand, execute and complete selection of suited components? | Likert-Scale  (1-5) |
| RQ1(g) | How much it was easy to understand, execute and complete component testing? | Likert-Scale  (1-5) |
| RQ1(h) | How much it was easy to understand, execute and complete test suit repository? | Likert-Scale  (1-5) |
| RQ1(i) | How much it was easy to understand, execute and complete documentation process? | Likert-Scale  (1-5) |
| RQ2(a) | How much effort did you expect to perform application requirements process? | Likert-Scale  (1-5) |
| RQ2(b) | How much effort did you expect to perform common reference architecture process? | Likert-Scale  (1-5) |
| RQ2(c) | How much effort did you expect to perform variations and commonalities identification process? | Likert-Scale  (1-5) |
| RQ2(d) | How much effort did you expect to perform component selection process? | Likert-Scale  (1-5) |
| RQ2(e) | How much effort did you expect to perform dependency evaluation process? | Likert-Scale  (1-5) |
| RQ2(f) | How much effort did you expect to perform selection of suited components process? | Likert-Scale  (1-5) |
| RQ2(g) | How much effort did you expect to perform component testing process? | Likert-Scale  (1-5) |
| RQ2(h) | How much effort did you expect to perform test suit repository process? | Likert-Scale  (1-5) |
| RQ2(i) | How much effort did you expect to perform documentation process? | Likert-Scale  (1-5) |
| RQ3(a) | How much quality do you think is increased using application selection of suited components for testing? | Likert-Scale  (1-5) |
| RQ3(b) | How much quality do you think is increased using component testing process? | Likert-Scale  (1-5) |
| RQ3(c) | How much quality do you think is increased using continuous updating of test suit repository? | Likert-Scale  (1-5) |
| RQ3(d) | How much quality do you think is increased dependency evaluation process? | Likert-Scale  (1-5) |
| RQ4(a) | how much complexity is reduced for the maintenance of the product using variations and commonalities identification? | Likert-Scale  (1-5) |
| RQ4(b) | how much complexity is reduced for the maintenance of the product using component selection? | Likert-Scale  (1-5) |
| RQ4(c) | how much complexity is reduced for the maintenance of the product using component selection dependency evaluation? | Likert-Scale  (1-5) |
| RQ4(d) | how much complexity is reduced for the maintenance of the product using selection of suited components? | Likert-Scale  (1-5) |
| RQ5(a) | How much improvement do you think is seen in product version management using dependency evaluation? | Likert-Scale  (1-5) |
| RQ5(b) | How much improvement do you think is seen in product version management using selection of suited components? | Likert-Scale  (1-5) |
| RQ5(c) | How much improvement do you think is seen in product version management using component testing? | Likert-Scale  (1-5) |
| RQ4(d) | How much improvement do you think is seen in product version management using documentation completion and updating? | Likert-Scale  (1-5) |
