# Supplementary material for: Towards a component-based system model to improve the quality of highly configurable systems
Source: PeerJ Comput Sci. 2022 Mar 7;8:e912. doi: 10.7717/peerj-cs.912 (PMC9044257; doi:10.7717/peerj-cs.912)
Supplement: Supplemental Information 3 [file peerj-cs-08-912-s003.docx]

**Appendix AE Product Line Engineering Process Report**

Table 0.1: Description template for analysis of agile SPLE model

| **Elements** | **Description** |
| --- | --- |
| Model Step ID | Text |
| Model Step Name | Text |
| Description | Text |
| Rational | Text |
| Possible Outcome of Step | Text |
| Objectives Achievement status | Yes/No/NA |

Table 0.2: Progress tracking report for analysis of agile SPLE model

| **Model Steps** | **Activities** | **Time (Hour)** | **Date** |
| --- | --- | --- | --- |
| Application Requirements | Time Spent on Applications Requirements Gathering |  |  |
|  | Total Time in solving problem and providing their solution for completing Application Requirements |  |  |
| Company Reference Architecture | Time Spent on Company Reference Architecture maintenance |  |  |
|  | Total Time in solving problem and providing their solution for completing Reference Architecture maintenance |  |  |
| Variation & Commonalities Identification | Time Spent on Variation & Commonalities Identification |  |  |
|  | Total Time in solving problem and providing their solution for completing Variation & Commonalities Identification |  |  |
| Component Selection | Time Spent on Component Selection |  |  |
|  | Total Time in solving problem and providing their solution for completing Component Selection |  |  |
| Dependency Evaluation | Time Spent on Dependency Evaluation |  |  |
|  | Total Time in solving problem and providing their solution for completing Dependency Evaluation |  |  |
| Selection of Suited Components | Time Spent on Selection of Suited Components |  |  |
|  | Total Time in solving problem and providing their solution for completing Selection of Suited Components |  |  |
| Component Testing | Time Spent on Component Testing |  |  |
|  | Total Time in solving problem and providing their solution for completing Component Testing |  |  |
| Test Suit Repository Updating | Time Spent on Test Suit Repository Updating |  |  |
|  | Total Time in solving problem and providing their solution for completing Test Suit Repository Updating |  |  |
| Documentation | Time Spent on Documentation maintenance |  |  |
|  | Total Time in solving problem and providing their solution for completing Documentation maintenance |  |  |
